# Post-Parotidectomy Reconstruction: A Systematic Review on Methods and Their Outcomes Regarding Overall Cosmesis

**DOI:** 10.51894/001c.144154

**Published:** 2025-09-30

**Authors:** Olivia C. Matz

**Affiliations:** 1 Otolaryngology McLaren Oakland

## 49

### INTRODUCTION

Despite its benefits, parotidectomy is not without consequences, as it can lead to functional deficits and aesthetic concerns. The salivary gland pathology, size, location and extent of disease may result in a superficial, deep, or total parotidectomy with or without facial nerve sacrifice and/or ipsilateral neck dissection.

### OBJECTIVE

Despite the array of reconstruction options available, there remains a notable gap in the literature regarding direct comparisons of these techniques regarding overall aesthetics. Our aim is to comprehensively evaluate the efficacy and outcomes of various parotidectomy defect reconstruction measures, particularly regarding overall aesthetic outcomes.

### METHODS

A literature review was conducted to identify articles related to parotidectomy defect reconstruction. The PRISMA checklist and statement recommendations were used as a guide during this review process. Data sources included PubMed, EBSCO, Scopus, and the Cochrane CENTRAL databases, which were initially queried March 31, 2023. Initial data obtained included literature from the years 1957 through 2023. This initial search combined key terms related to the parotidectomy procedure, salivary gland, reconstruction, and goals (aesthetics).

### RESULTS

99 articles met inclusion criteria. The most utilized reconstructive procedures include sternocleidomastoid (n=218, 36 inferiorly based) and superficial musculo-aponeurotic system (n=339, many paired with adjuvant procedures) flaps, and dermal fat grafts (n=402). Most patients were satisfied with their cosmetic outcome regarding facial contour, however the variability of study designs, limited and non-standardized measurements to assess patient satisfaction, and often anecdotal descriptions from authors prevent performance of meta-analysis, currently.

### DISCUSSION/CONCLUSIONS

The most performed reconstruction method employed for parotidectomy defects was the free dermal fat graft followed by SMAS and SCM flaps. Many studies that detailed patient satisfaction designed their surveys with different terms in the measure of satisfaction. While many publications included one or more pictures of patients before and after surgery, authors bias in the selection of cases they publish may exist, such as selecting those with the best outcomes.

This systematic review explores various approaches to post-parotidectomy reconstruction and offers insights into the selection of reconstruction methods, enhancing our understanding of their effectiveness and patient satisfaction.

